# Beta-adrenergic receptors support attention to extinction learning that occurs in the absence, but not the presence, of a context change

**DOI:** 10.3389/fnbeh.2015.00125

**Published:** 2015-05-27

**Authors:** Marion Agnès Emma André, Oliver T. Wolf, Denise Manahan-Vaughan

**Affiliations:** ^1^International Graduate School for Neuroscience, Ruhr University BochumBochum, Germany; ^2^Faculty of Psychology, Department of Cognitive Psychology, Ruhr University BochumBochum, Germany; ^3^Medical Faculty, Department of Neurophysiology, Ruhr University BochumBochum, Germany

**Keywords:** extinction learning, noradrenaline, rodent, spatial learning, beta-blocker, hippocampus, propranolol

## Abstract

The noradrenergic (NA)-system is an important regulator of cognitive function. It contributes to extinction learning (EL), and in disorders where EL is impaired NA-dysfunction has been postulated. We explored whether NA acting on beta-adrenergic-receptors (β-AR), regulates EL that depends on context, but is not fear-associated. We assessed behavior in an “AAA” or “ABA” paradigm: rats were trained for 3 days in a T-maze (context-A) to learn that a reward is consistently found in the goal arm, despite low reward probability. This was followed on day 4 by EL (unrewarded), whereby in the ABA-paradigm, EL was reinforced by a context change (B), and in the AAA-paradigm, no context change occurred. On day 5, re-exposure to the A-context (unrewarded) occurred. Typically, in control “AAA” animals EL occurred on day 4 that progressed further on day 5. In control “ABA” animals, EL also occurred on day 4, followed by renewal of the previously learned (A) behavior on day 5, that was succeeded (on day 5) by extinction of this behavior, as the animals realised that no food reward would be given. Treatment with the β-AR-antagonist, propranolol, prior to EL on day 4, impaired EL in the AAA-paradigm. In the “ABA” paradigm, antagonist treatment on day 4, had no effect on extinction that was reinforced by a context change (B). Furthermore, β-AR-antagonism prior to *renewal* testing (on day 5) in the ABA-paradigm, resulted in normal renewal behavior, although subsequent extinction of responses during day 5 was prevented by the antagonist. Thus, under both treatment conditions, β-AR-antagonism prevented extinction of the behavior learned in the “A” context. β-AR-blockade during an overt context change did not prevent EL, whereas β-AR were required for EL in an unchanging context. These data suggest that β-AR may support EL by reinforcing attention towards relevant changes in the previously learned experience, and that this process supports extinction learning in constant-context conditions.

## Introduction

Arousal and attention are key factors in effective learning behavior. Attending to experience both facilitates and expedites learning, and one of the key neuromodulators that regulates this process is noradrenaline (Crow, 1968; Kety, 1970, 1972; Aston-Jones and Bloom, 1981a,b; Sara and Segal, 1991). Attending to experience is also a key element in the process of extinction learning, whereby an individual learns that a prior learned experience no longer fulfills its learned function, or is no longer relevant. In biological terms, this means that the response to a conditioned stimulus (CS) declines when the stimulus is presented without reinforcement. In cognitive terms it means learning, for example, that the neighbor’s house is no longer frightening, because the dog that bit you has been removed, or because it subsequently desists from biting you.

Extinction learning can thus be expected to occur under two possible conditions: the removal of the neighbor’s dog comprises a context change, and substantial evidence exists that this strongly facilitates extinction (Bouton, 2004), whereby the circumstance whereby the neighbors dog remains in residence but never bites you again, amounts to extinction learning in the *absence* of a context change. Understanding the mechanisms that facilitate extinction is an important goal in understanding how extinction occurs at the cellular level, and in identifying strategies to optimise extinction. The noradrenergic (NA) system has been subjected to considerable attention in this regard, due to its postulated role in impaired extinction learning, for example, in post-traumatic stress disorder (Taylor and Raskind, 2002; Peskind et al., 2003; Griffith, 2005). Although it is clear that NA modulation of the amygdala plays a very important role in the learning and extinction of emotive memories mediated by the amygdala (Debiec and Ledoux, 2004; Roozendaal and McGaugh, 2011), much less is understood about the role of the NA system in extinction learning processes that are supported by the hippocampus. The hippocampus is involved in the assimilation and retrieval of context during novel extinction learning as well as during recall of context-dependent fear extinction (Good and Honey, 1991; Hobin et al., 2006; de Carvalho Myskiw et al., 2014; Portugal et al., 2014; Tan et al., 2014), and as well as during associative learning in humans (Lissek et al., 2013). It is also strongly implicated in context-dependent extinction in the absence of fear-reinforcement (Wiescholleck et al., 2014). Furthermore, the dorsal hippocampus contributes to the renewal of the conditioned response following fear extinction (Ji and Maren, 2005).

Current reports suggest that is that the hippocampus is particularly important for context-dependent extinction (Kalisch et al., 2006). Most studies have examined this with regard to fear-extinction (Alvarez et al., 2008; Lang et al., 2009; Maren et al., 2013), but recently, it was demonstrated that extinction learning in an appetitive context is also likely to involve the hippocampus (André et al., 2015). In rodents, context-dependent spatial learning, as well as hippocampal synaptic plasticity that is triggered by spatial learning, is supported by β-adrenergic receptors (Kemp and Manahan-Vaughan, 2008; Hagena and Manahan-Vaughan, 2012; Goh and Manahan-Vaughan, 2013). Furthermore, object-context learning triggers β-adrenergic receptor-dependent synaptic plasticity in the hippocampus (Kemp and Manahan-Vaughan, 2008; Hagena and Manahan-Vaughan, 2012; Goh and Manahan-Vaughan, 2013; Hansen and Manahan-Vaughan, 2014). We therefore postulated that NA modulation via activation of β-adrenergic receptors may be important for extinction learning of an associative spatial learning task. To test this possibility, we examined whether β-adrenergic receptors contribute to extinction learning in a T-maze task, when the context remains consistent, or when extinction is facilitated by a context change.

## Materials and Methods

The present study was carried out in accordance with the European Communities Council Directive of September 22nd, 2010 (2010/63/EU) for care of laboratory animals. All experiments were performed according to the guidelines of the German Animal Protection Law and were approved by the North Rhine-Westphalia State Authority (Bezirksamt, Arnsberg). All efforts were made to reduce the number of animals used.

### Animals

Male Wistar rats (7–8 weeks old) underwent implantation of guide cannulae, whilst under anesthesia (52 mg/kg sodium pentobarbital via intraperitoneal (i.p.) injection), as described previously (Manahan-Vaughan, 1997). One cannula was implanted into the lateral cerebral ventricle of each hemisphere (0.5 mm posterior to bregma, 1.6 mm lateral to the midline; size: 5.6 mm length, 0.8 mm diameter, 4.5 mm depth).

Animals were allowed 2 weeks to recover, before any behavioral experiment took place. They were housed singly and maintained on a 12-h light/12-h dark cycle with food and water *ad libitum*.

Two days prior to commencing the behavioral training, the rats were weighed and food access was reduced to result in a consistent body weight of 85% relative to the animal’s weight immediately prior to starting the study. During the habituation phase, the animals were handled individually for 20 min per day.

### T-maze and Extinction Task

Experiments were conducted in a T-maze that comprised a starting box (25 × 20 cm) that was separated from the main corridor (100 × 20 cm) by a sliding door and two side corridors (40 × 10 cm) positioned perpendicular to the other end of the main corridor, as described previously (Wiescholleck et al., 2014). The walls were 40 cm high. At the end of each arm, at a distance of 1 cm from the end wall, a small round cup was placed on the floor equidistant from the walls, in which a reward could be placed. The reward could not be seen from a distance.

The context of the maze was changed in three ways, as described previously (Wiescholleck et al., 2014): (1) the plastic floor of the maze could be exchanged. Typical floor patterns comprised zebra stripes, checkered patterns, or geometric lines; (2) at the end of the 2 arms odors were placed that could be exchanged—1 μl of almond or vanilla (food aroma, Dr. Oetker, Bielefeld, Germany) was used; (3) extra-maze cue cards were used that could also be exchanged (Din A5 white paper with a black cross or a black square). These were placed 40 cm above the end of the main corridor.

On each experiment day, rats participated in a learning session that comprised 20 consecutive trials, that were split into two data blocks (1st 10, 2nd 10 trials) for analysis purposes (see below). The trial commenced with the opening of the door to the starting box, whereupon the animal entered the maze. The trial concluded when the animal entered an arm of the T-maze or when a specific time-limit (see below) had elapsed in the absence of arm entry. Animals learned to locate a food pellet (Dustless Precision Pellets 45 mg, BioServ, USA) that was placed at the end of a predetermined arm. This “correct” arm remained constant for a given animal during the training days. The floor and odor context were also kept constant during this time. On days 1 through 3, the reward probability was reduced in a stepwise manner from 100% to 25% to augment extinction resistance, as described previously (André et al., 2015). In conjunction with the reward probability reduction, the time limit for reaching the arm was also reduced from 2 min to 30 s Learning criterion was deemed to be acheived when the animal had successfully entered the correct arm on 8 of the final 10 trials of a given experiment day. Animals that failed to reach criterion by day 3 were excluded from the remainder of the study and their data from days 1–3 were not included in the analysis.

On day 4, extinction learning was assessed, whereupon the animals participated in 20 trials, during which no reward was present at any time. One day later (day 5), renewal (RN) was assessed by re-introducing the animal to the original T-maze (A) context for 20 trials with no food reward.

One animal cohort was tested in an AAA paradigm, where all trials (days 1–5) were conducted in the same context. A second cohort was assessed in an ABA paradigm, in which training was conducted in context A while the extinction session was conducted in context B, whereby the context (floor, odor and cue card) had been changed (André et al., 2015).

On day 5, animals (in both cohorts) were returned to the “A” context (in the absence of food reward). Typically, further extinction occurs under control conditions in the AAA group, whereas renewal of the behavior learned in the A context (1st 10 trials) followed by extinction of this behavior due to the lack of food reward (2nd 10 trials) occurs in the ABA groups (Wiescholleck et al., 2014; André et al., 2015).

### Analysis of Decision Time

Decision-time typically declines, in close alignment with the increase in choice confidence on the part of the animal, during the gradual acquisition of the T-maze task (Luce, 1986; Avila and Lin, 2014; André et al., 2015). We evaluated this by recording the time required to leave the start box and reach the arm chosen by the animal. We evaluated this for every choice (incorrect and correct choices). By this means we obtained a measure of the confidence of the animal as to which arm was the correct choice (André et al., 2015).

### Pharmacological Treatment

The β-adrenergic receptor antagonist, propranolol (Tocris Bioscience, Bristol, UK), was dissolved in 0.9% NaCl in a dosage of 2 μg/5 μl. This dose does not affect basal synaptic transmission in the hippocampus (Kemp and Manahan-Vaughan, 2008). The bilateral guide cannulae were inserted, and after ca. 5 min, a 5 μl solution volume was injected at a rate of 1 μl/min. The cannulae were left in place for a minimum of 5 min before removal (André et al., 2015). Propranolol, or vehicle, was given 30 min prior to the first trial of the extinction day (day 4) in the AAA and ABA paradigms. In a separate experiment with a third animal cohort, propranolol, or vehicle, was applied 30 min before the 1st trial before renewal testing on day 5 in the ABA group.

### Data Analysis

Correct answers were defined as trials in which the animal moved first to the target arm. Each 20-trial session was divided into two sets of 10 trials (first 10 and last 10 trials), as described previously (André et al., 2015). The time required to reach the end of the first arm visited was calculated for each trial.

To analyse decision time, the time taken by the animal to move from the departure area in the T-Maze to its arm of choice was recorded for each trial, and data were segregated into 4 sets of 5 trials for each day, of which the times were averaged (André et al., 2015).

Data were analyzed by means of a multifactorial analysis of variance (ANOVA) with repeated-measures including 2 within-subject factors (Day and Session) and 2 between-group factors (Treatment and Experimental Design). Differences between trial blocks or between trials days of a specific group (control or propranolol-treated animals) were assessed using Bonferroni *post hoc* tests. Except where “ANOVA” is mentioned explicitly, all *p* values in the results section correspond to values determined from the Bonferroni test. The level of significance was set at *p < 0.05*.

## Results

### Extinction in the AAA Paradigm is Prevented by Antagonism of β-Adrenergic Receptors

**Figure 1:**
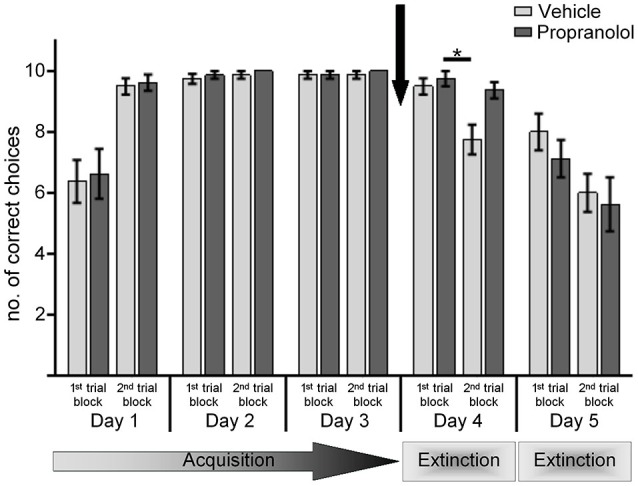
**Antagonism of β-adrenergic receptors prevents extinction learning in the AAA paradigm**. Animals underwent 20 contiguous trials per day of training in the AAA paradigm. Bar charts represent the number of correct arm choices in the first and second set of 10 trials on each test day. Animals participated in 3 days of acquisition training in the AAA paradigm, ending on day 3 with a 25% reward probability. Control animals were treated with vehicle prior to re-exposure to the context on day 4, in the absence of reward. Here, by the 2nd set of 10 trials significant extinction was evident. Upon return to the same context on day 5 (without reward) a further extinction of the learned conditioned stimulus (CS)-US response was shown. Treatment of animals, with the β-adrenergic receptor antagonist propranolol, before re-exposure to the A context in the absence of reward on day 4, significantly impaired extinction learning. A return to the same context on day 5 resulted in extinction of the learned response. An asterisk indicates a significant effect of at least *p* < 0.01 between the trials indicated by the bar. The arrow signifies the time of antagonist/vehicle-injection.

During the first 3 experiment days, animals learned to take a constant turn (e.g., left) in a T-Maze to obtain a food reward, whereby reward probability was systematically reduced to 25% by the last trial block of day 3. A significant difference in performance was evident between day 1 and day 2 (Figure 1), reflecting successful acquisition of the task (ANOVA: for animals subsequently treated with vehicle, *p* < 0.001, *n* = 8; for animals subsequently treated with propranolol, *p* < 0.001, *n* = 8). No significant difference was evident in performance within the first and second 10 trial block on day 3, at which point, learning criterion had been reached (Figure 1). No significant difference in the animals’ performance was evident on days 1, 2 or 3 when the two animals cohorts were compared (*F*_(1.06,13.783)_ = 0.07; *p* = 0.81).

On day 4 and 5 the animals were returned to the same context but received no reward (AAA paradigm). Thirty minutes prior to commencing the first trial on day 4, animals were treated with either the β-adrenergic receptor antagonist, propranolol (*n* = 8), or vehicle (*n* = 8).

In both treatment groups, performance levels were equivalent in the 1st ten trials of day 4 (*p* > 0.001). Furthermore, performance levels were equivalent during the 1st ten trials of day 4 compared to the last ten trials of day 3 ANOVA: for control animals, *p* > 0.001; for propranolol-animals, *p* > 0.001, *n* = 8).

Differences became apparent in the 2nd trial block on day 4, however (Figure 1). Here, vehicle-treated animals exhibited significant extinction of the learned response when performance in the 1st trial block on day 4 was compared to performance in the 2nd trial block (*p* < 0.001). In contrast, propranolol-treated animals failed to show this extinction effect. Here, performance in the 2nd trial block was equivalent to performance in the 1st trial block (*p* > 0.001). Furthermore, the performance of the vehicle and propranolol-treated animals during the first and second trial blocks on day 4 was significantly different (ANOVA: *F*_(1,14)_ = 11.486; *p* = 0.005). Thus, extinction in the AAA paradigm, in the absence of a context change, is impaired by prior treatment with a β-adrenergic receptor antagonist.

On day 5, animals were re-exposed to the same context in the absence of reward. Here, performance in vehicle-treated animals was equivalent in the 1st set of trials compared to performance in their last trial block on day 4 (*p* = 0.514). Extinction continued during the trials, with correct arm choices in the 2nd trial block on day 5 being significantly poorer than in the 1st trial block *p* < 0.001).

Effects were similar in the animals that had been treated on day 4 with propranolol. Here, their performance during the 1st and 2nd trial blocks on day 5 were equivalent to vehicle-treated controls (ANOVA: *F*_(1,14)_ = 1.112; *p* = 0.311), although their performance in the 1st 10 trials was significantly reduced compared to their performance in the last trial block on day 4 (*p* < 0.002). Thus, in the absence of propranolol, extinction learning was equivalent.

These data suggest that, antagonism of β-adrenergic receptors impair extinction learning in the absence of a context change.

### Extinction in the ABA Paradigm is not Prevented by Antagonism of β-Adrenergic Receptors

A context change in the T-maze paradigm has been shown to facilitate extinction (Wiescholleck et al., 2014). Here, the protocol was identical to the AAA paradigm described above, except that on day 4 (“B” context) the floor pattern was changed, as were the odor-related and extramural cues. On day 5, the animals were re-exposed to the “A” context that they had experienced on days 1–3. On days 4 and 5, no reward was given, as was the case for the AAA paradigm. Thirty minutes prior to commencing the first trial on day 4, animals were treated with either propranolol (*n* = 10) or vehicle (*n* = 10).

**Figure 2:**
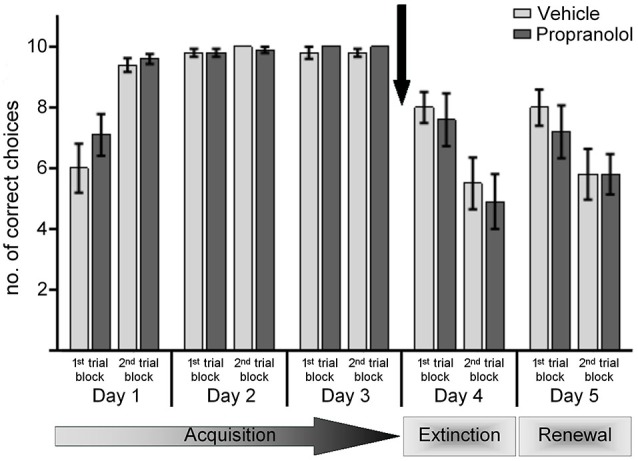
**Antagonism of β-adrenergic receptors before the extinction trials in a new context (ABA paradigm) does not prevent extinction learning**. Control animals were treated with vehicle prior to exposure to the novel context “B” on day 4, in the absence of reward. Here, by the 2nd set of 10 trials significant extinction was evident that was also significantly better than extinction learning under the same conditions in the “A” context. Upon return to the learning context “A” on day 5 (without reward) an initial recovery (renewal) of the learned CS-US response was evident in the 1st set of 10 trials that was followed by significant extinction of the CS-US response. Treatment of animals with the β-adrenergic receptor antagonist, propranolol, before novel exposure to the “B” context in the absence of reward on day 4, had no effect on extinction learning. A return to the learning context “A” on day 5 resulted in renewal of the learned CS-US response (in the 1st 10 trials), that was followed by an extinction of this learned response during the last 10 trials of the day. Responses were equivalent to this observed in control animals. The arrow signifies the time of antagonist/vehicle-injection.

In vehicle-treated animals, extinction occurred on day 4 that was significantly better than extinction effects in the AAA paradigm (Figure 2) (*p* < 0.029), in line with previous results (Wiescholleck et al., 2014). Performance in the second trial block on day 4, was significantly weaker than in the first trial block (*p* < 0.006) indicating that significant extinction had occurred.

Treatment of animals with propranolol 30 min prior to entering the “B” context on day 4, had no significant effect on extinction learning (Figure 2): the performance of the animals was equivalent to that seen in controls (ANOVA: *F*_(1,18)_ = 0.258; *p* = 0.618).

These data suggest that antagonism of β-adrenergic receptors does not influence extinction learning that is supported by a change of context.

Re-exposure to context “A” on day 5 elicited significant renewal effects in both animal groups (Figure 2). Thus, a comparison of the last trial block on day 4 with the 1st trial block on day 5, revealed a significantly improved correct choice performance in both the vehicle-treated animals (*p* < 0.05), and in animals that had been treated with propranolol on day 4 (*p* < 0.05). Thus, β-adrenergic receptor-antagonism does not affect renewal of the experience learned in the “A” context.

In both animal groups, extinction of this renewal effect became evident during the second set of 10 trials on day 5 (Figure 2) (*p* < 0.001, 1st vs. 2nd 10 trials, for both cohorts). No significant effect was evident when performance on day 5 was compared in the control and propranolol-treated animals (ANOVA: *F*_(1,18)_ = 0.196; *p* = 0.663).

### Antagonism of β-Adrenergic Receptors Prior to the Renewal Test in the ABA Paradigm has no Effect on Renewal but Prevents Extinction of the Old Context

The lack of effect of the β-adrenergic receptor-antagonist could derive from the fact that by 24 h after drug administration, its biological titre is so low as to no longer effectively block β-adrenergic receptors. This likelihood is supported by the finding that on day 5, in the AAA paradigm, no extinction impairment occurs. Thus, to clarify if β-adrenergic receptor antagonism has no bearing on renewal, we applied the antagonist 30 min before trial-begin *on day 5* in the ABA paradigm.

**Figure 3:**
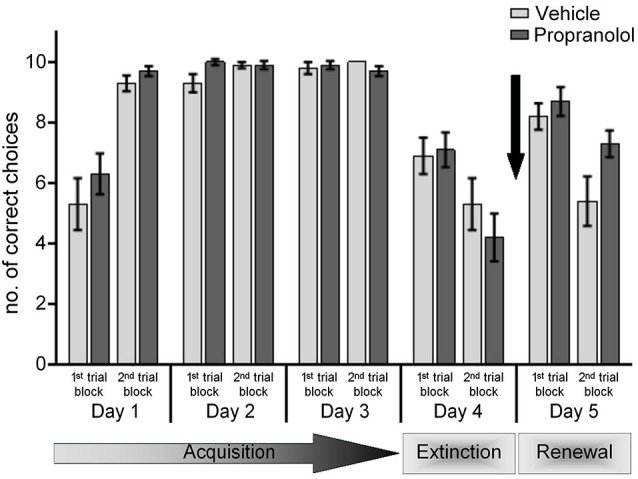
**Antagonism of β-adrenergic receptors before renewal in the ABA paradigm has no effect**. On day 5, of the ABA paradigm, before the exposure to the learned “A” context in the absence of reward, control animals were treated with vehicle. During the first 10 trials, there was a recovery of the learned response that was followed by its extinction during the last 10 trials. Treatment of animals with the β-adrenergic receptor antagonist, propranolol, before the re-exposure to the learned context “A” on day 5, did not have any effect on the renewal effect (1st set of trials), but significantly inhibited subsequent extinction (2nd set of trials). The arrow signifies the time of antagonist/vehicle-injection.

Under these circumstances, renewal was also equivalent in vehicle-treated (*n* = 10) and propranolol-treated animals (*n* = 10) (Figure 3). Here, we observed a significant renewal of the response learned in context “A” in both vehicle-injected and propranolol-treated animals (*p* < 0.001, for both groups), when performance in the 1st trial block on day 5 was compared to performance in the last trial block on day 4. Renewal effects were also equivalent in both animal groups (ANOVA: *F*_(1,18)_ = 0.181; *p* = 0.676). Strikingly, although vehicle-treated animals exhibited significant extinction in the last trial block of day 5 (*p* < 0.001, compared to the 1st trial block on day 5), extinction was impaired in the propranolol-treated group (*p = 0.108*, 1st vs. 2nd trial block, day 5). Furthermore, the performance of the control and propranolol-treated animals was also significantly different from one another during the 2nd (extinction) trial block on day 5 (ANOVA: *F*_(1,18)_ = 5.469; *p* = 0.032).

The data confirm that renewal is unaffected by β-adrenergic receptor-antagonism. The data further indicate that β-adrenergic receptors are required for extinction (in the AAA paradigm) and (re-)extinction in the “A” context within the ABA paradigm. In other words β-adrenergic receptors are only required when extinction learning takes place in the context in which the original learning occurred.

### Antagonism of β-Adrenergic Receptors has no Effect on Decision-Time in the “ABA” Paradigm but Improves Decision Time during Extinction Learning in the “AAA” Paradigm

**Figure 4:**
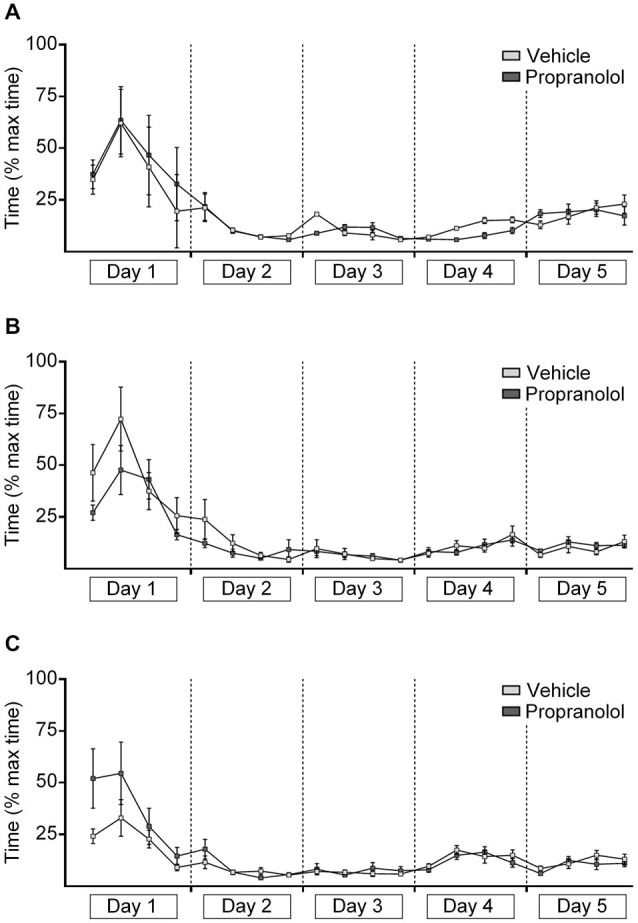
**Antagonism of β-adrenergic receptors has no effect on decision-time in the “ABA” paradigm, but improves decision time during extinction learning in the “AAA” paradigm**. The graphs represent the amount of time that was needed to reach the end of an arm (both correct and incorrect choices) after door-opening. For each day, the time for 5 contiguous trials was averaged (i.e., 4 time-points per day “injection prior to extinction on day 4” are shown). Decision-times recorded in the AAA paradigm (**A**, injection prior to extinction on day 4), the ABA paradigm (injection pre-extinction on day 4) **(B)** and the ABA paradigm paradigm (injection prior to the renewal trials on day 5) **(C)** are shown. During learning of the task, the time needed to reach the end of an arm steadily decreased while the correct answers increased, until a basal level of correct answers was reached on day 3 that reflected animals reaching the 80% criterion of correct arm choices. During the extinction and renewal trials, the decision-time increased in parallel with the decrease of correct choices. Propranolol, had no effect on decision time during extinction learning on day 4 **(B)**, and renewal on day 5 **(C)** in the “ABA” paradigm. Decision time during extinction learning was improved in the presence of propranolol in the “AAA” paradigm **(A)**.

When animals begin to acquire the task, the decision-time decreases in conjunction with an improvement in correct choices (Luce, 1986; Avila and Lin, 2014). Conversely, during extinction learning, decision-time typically increases if an attrition in the number of correct arm choices occurs (André et al., 2015). The latter situation was the case for vehicle-treated and propranolol-treated animals in the periods encompassing day 1 and day 3 (task acquisition), under all conditions tested (Figure 4). In other words decision-time steadily decreased as the animals acquired the task and reached the learning criterion.

In contrast, in the AAA paradigm, in vehicle-treated animals (*n* = 8) extinction learning on days 4 and 5 was paralleled by a steady increase in decision-time (*p* < 0.001) (Figure 4A), reflecting the increasing insecurity of the animals as to which arm to choose. Decision-time was equivalent on day 5 in both vehicle-treated and propranolol-treated animals (*n* = 8). However, a direct comparison of decision time during day 4 revealed significantly faster decision times in propranolol-treated animals (*F*_(1,14)_ = 11.523; *p* = 0.004).

In the ABA paradigm during renewal (day 5), decision-time continued to decrease in both animals group (each *n* = 10, Figures 4B,C), whereby decision time increased slightly (n.s.) in the time-frame of the 2nd set of trial blocks, whereupon extinction had occurred.

Antagonism of β-adrenergic receptors using propranolol prior to extinction, did not affect the overall trend towards an increase in decision-time across days 4 and 5 in the AAA paradigm (Figure 4A), (*F*_(1,14)_ = 2.148; *p* = 0.165) (Figure 4A), or on performance in the ABA paradigm where propranolol was given on day 4 (Figure 4B), (*F*_(1,18)_ = 0.02; *p* = 0.89).

The injection of propranolol prior to the renewal trials in the ABA protocol also didn’t influence the decision time (*F*_(1,18)_ = 1.154; *p* = 0.297, each *n* = 10) (Figure 4C). Thus, treatment with propranolol did not impair the choice-making confidence of the animals, suggesting that consolidation of the extinction experience was not influenced by propranolol treatment. The fact that propranolol improved decision-times during extinction in the “AAA” paradigm, suggests that the impairments of extinction observed under these conditions (Figure 1) may relate to a reduction in attention.

## Discussion

The data of this study indicate that when extinction learning occurs under non-emotive circumstances, release of noradrenaline and subsequent activation of β-adrenergic receptors is a critical factor. We observed that antagonism of β-adrenergic receptors prevents extinction learning if the context remains constant. In contrast, extinction learning is unaffected by β-adrenergic receptor–antagonism, if extinction is reinforced by a context change. This suggests that the β-adrenergic receptor is required for extinction learning of a consolidated learned experience, whereby it supports attention to the absence of the CS, and the subsequent adaptation in behavior that results. Where attention to the absent CS is reinforced by a context change, support of extinction by β-adrenergic receptors becomes redundant, presumably because the increased arousal triggered by the context change mediates activation of additional neuromodulatory systems that support and reinforce extinction (e.g., dopamine or corticosterone). This likelihood is reinforced by the finding that renewal of the learned experience (in the “A” context, on day 5), following extinction in the “B” context, is unaffected by β-adrenergic receptor–antagonism (applied prior to testing on day 5), whereas the subsequent (re)-extinction of this behavior is prevented. The finding that decision-time is unaffected by β-adrenergic receptor antagonism in the ABA contexts, but is improved during extinction learning in the AAA context, suggests that it is not learning *per se*, but rather attention to the salient elements of the experience that is modulated by β-adrenergic receptors during extinction.

Although many studies have addressed the role of noradrenaline and β-adrenergic receptors in extinction of aversive experience (Cain et al., 2004), little is known about its role in extinction of appetitive memory (Mueller and Cahill, 2010), as was the focus of the current study. A role for noradrenaline in both memory consolidation has been reported (Quirarte et al., 1997; Roozendaal et al., 2002). Furthermore, noradrenaline is involved in fear extinction consolidation processes (Ouyang and Thomas, 2005; but see also: Lonsdorf et al., 2014). In the present study we did not see such an effect with regard to consolidation of extinction of appetitive memory, at least in terms of the involvement of β-adrenergic receptors: renewal of the learned response was unaffected by treatment with a β-adrenergic receptor antagonist prior to extinction learning. Two possible explanations spring to mind: on the one hand, the studies, where noradrenaline involvement in extinction consolidation was reported, were predominantly conducted under the conditions of fear extinction (Mueller and Cahill, 2010) and were particularly related to context-dependent extinction (Ouyang and Thomas, 2005), leading to the proposal that noradrenaline release onto β-adrenergic receptors is particularly relevant for context-dependent fear extinction (Mueller and Cahill, 2010). On the other hand, we cannot exclude that consolidation of extinction learning depends on the activation of adrenergic receptors other than the β-adrenergic receptors. In fact, evidence exists that different adrenergic receptors may play different roles in the regulation of extinction learning, and this may relate to their relative sensitivity to noradrenaline and the signaling pathways to which they couple. For example, although we observed that β-adrenergic receptor antagonism prevents extinction learning in an unchanged context, others have reported that antagonism of α2-adrenergic receptors *enhances* extinction in an unchanged context (Morris and Bouton, 2007). This may relate to the differences in the paradigms used (non-fearful memory vs. conditioned-fear memory), and thus, to the relative release of noradrenaline from the locus coeruleus triggered by these different experiences (Bouret and Sara, 2004; Sara, 2009), as well as and to differences in receptor-sensitivity to noradrenaline (Ahlquist, 1948; Molinoff, 1984). Furthermore, whereas β-adrenergic receptors are positively coupled to adenylyl cyclase (Strader et al., 1989) and promote insertion of the AMPA-receptor subunit, GluA1/GluR1, into the postsynapse (Joiner et al., 2010), α2-adrenergic receptors are negatively coupled to adenylyl cyclase and suppress activity of voltage-activated Ca^2+^-channels and activate receptor-operated K^+^-channels (Limbird, 1988). Thus, these receptors can be expected to mediate opposing effects on neuronal function. Nonetheless, our data suggest that β-adrenergic receptor activation is not required for consolidation of extinction learning. However, we saw clear effects of β-adrenergic receptor antagonism on extinction learning in the absence of a context change. This suggests that activation of β-adrenergic receptors may be required to support attentional focus on the CS to enable effective extinction learning under these circumstances.

In the central nervous system, noradrenaline is released from afferent fibers that originated in the locus coeruleus, the firing of which increases in response to novelty (Sara, 2009), and a variety of behaviorally relevant stimuli such as unexpected events, threats, reward or fear (Sara and Bouret, 2012). The degree of activity of the locus coeruleus is graded according to the saliency of the experience, whereby the slow tonic changes in firing rates that accompany fluctuations in arousal state, can rapidly change into burst firing upon exposure to noxious stimuli, for example (Valentino and Van Bockstaele, 2008). The locus coeruleus also exhibits a very specific activity profile in response to conditioned stimuli, whereby firing can become persistent and intensify if a stimulus is followed by a salient event (Aston-Jones et al., 1994; Sara et al., 1994; Bouret and Sara, 2004), and firing is also triggered during extinction of appetitive and aversive learning (Sara and Segal, 1991). Furthermore, emotionally arousing experiences reinforce the acquisition emotional experiences via activation of β-adrenergic receptors (Liang et al., 1986; Cahill et al., 1994; Ji et al., 2003; Grillon et al., 2004). In an interesting parallel to the ability of the locus coeruleus to engage in noradrenaline release that is graded according to the saliency of the experience, the hippocampus exhibits graded sensitivity to NA (Loy et al., 1980). The dentate gyrus is the most sensitive, followed by the CA3 region and the CA1 region (Loy et al., 1980). The hippocampus engages in the very precise sorting of learned associative experiences, such that the discrimination of stored experiences from novel similar experiences occurs (pattern separation), presumably at the level of the dentate gyrus (Kesner, 2013a,b). By contrast, retrieval of associative memories based on exposure to a fragment of that memory (pattern completion) is enabled by the CA3 and possibly the CA1 region (Kesner, 2013a,b). In the present study, we saw that extinction learning in the absence of a context change is supported by β-adrenergic receptors. This process is arguably supported by pattern separation mechanisms in the hippocampus. Learning under these conditions would not be expected to trigger intense noradrenaline release from the locus coeruleus, but this may be sufficient to selectively support information processing and pattern separation in the dentate gyrus.

Many of the effects on cognition and synaptic plasticity of noradrenaline, released from the locus coeruleus, are mediated by β-adrenergic receptors, (Lemon et al., 2009; Lemon and Manahan-Vaughan, 2012; Goh and Manahan-Vaughan, 2013; Hansen and Manahan-Vaughan, 2014), and the T-maze task we used in our study, because included both spatial and context-dependent learning elements, is likely to recruit hippocampal information encoding. For this reason we hypothesized that β-adrenergic receptors would be required for extinction learning in this task. Thus, it was surprising to find that antagonism of β-adrenergic receptors only prevented extinction learning in the AAA paradigm, given the fact that the change in context during extinction learning in the ABA paradigm would be expected to elicit a higher level of locus coeruleus firing and thus, of noradrenaline release. Extinction of context “A” was impaired when propranolol was applied prior to extinction learning on day 4 (AAA paradigm), and when applied prior to re-exposure to the (unrewarded) “A” context on day 5 (ABA paradigm), suggesting that the robustness of this effect was not compromised by the context-dependent extinction event on day 4 in the “ABA” paradigm. We did not see an effect in extinction in the “A” context on day 5, when propranolol was given prior to extinction learning in context “B” on day 4, however. We propose that this is because propranolol is rapidly metabolized from the animals’ system (Bargar et al., 1983; Baughman et al., 2009) and few or no β-adrenergic receptors remained under the influence of the antagonist when behavior was tested 24 h after the antagonist had been applied. Taken together, our data suggest that β-adrenergic receptor activation is an important component for extinction learning in the absence of a context change.

This is not the case, however, for context-dependent extinction. The change of context facilitated extinction in control animals, and this effect was not hindered by antagonism of β-adrenergic receptors. One possibility is that the context change promotes a more intense release of noradrenaline from the locus coeruleus that activates β-adrenergic receptors in the hippocampal CA regions (Loy et al., 1980) and promotes the novel encoding of this new associative experience. Another possibility is that under conditions of increased arousal during the context change, dopamine that is released from the locus coeruleus (Lemon and Manahan-Vaughan, 2012; Smith and Greene, 2012) serves to reinforce the extinction learning process and compensated for the absence of β-adrenergic receptors (that occurred under the experimental conditions of the present study). In contrast, under conditions where no context change accompanied extinction learning, arousal levels can be expected to be comparatively lower, and learning under these conditions was tightly dependent on noradrenaline acting on β-adrenergic receptors. This possibility is supported by observations that depletion of noradrenaline impairs extinction learning of appetitive behavior (Mason and Iversen, 1975, 1978; Mason, 1979; McGaugh, 2002). It may also be the case that a more intense NA release was stimulated by the context change that was not overcome by the antagonist dose used. However, this seems less likely, because treatment of an animal cohort prior to re-exposure to the “A” context, following successful extinction learning in the “B” context, failed to prevent renewal but significantly prevented subsequent re-extinction of the behavior learned in the “A” context.

It was striking that following inhibition of extinction in day 4 in the AAA context (following prior treatment with propranolol), renewal behavior occurred in the “A” context on day 5. We believe this effect adds support to our interpretation that β-adrenergic receptor antagonism affected attention but not learning *per se*. If learning had been impaired by the antagonist, a further, at least initial, suppression of extinction would have been expected on day 5: the animals had not learned (on day 4) that the “A” context can no longer be associated with a reward and thus, do not persevere to search for a reward in this context. Our animals showed renewal behavior, however, that refutes this possibility. If attention, *and not*, learning was affected by the antagonist, then the animal could be expected to fail to notice (on day 4) that the selected arm had previously been entered without reward success. This is not implausible, bearing in mind that reward probability had been reduced to 25% on day 3. Cumulatively, during day 4, the animal could still learn that in total, no food reward at all had been found during the 20 trials, but not bring this behavior into association with the previously learned CS-US response. Under these conditions, the animal would be expected to show normal initial renewal behavior on day 5. This was indeed the case in the present study.

Propranolol did not affect decision-time in the ABA paradigm, but in the AAA paradigm, decision times were slightly, but nonetheless, significantly better in the presence of propranolol during extinction learning on day 4. Despite this, extinction was impaired in the AAA paradigm in the presence of the β-adrenergic receptor antagonist. This further suggests that attention was undermined, and the animals failed to notice that the 25% reward probability had decreased to 0%. Blocking β-adrenergic receptors impairs rodent and human performances in attentional tasks (Hahn and Stolerman, 2005; de Martino et al., 2008). Furthermore, noradrenaline release from the locus coeruleus serves to enhance neuronal responses towards discrete stimuli and thereby to increase the signal-to-noise ratio (Woodward et al., 1979; Sara, 1985; Servan-Schreiber et al., 1990; Lemon and Manahan-Vaughan, 2012). Attentional set-shifting is supported by noradrenaline acting on the medial prefrontal cortex (Lapiz and Morilak, 2006; Tait et al., 2007; McGaughy et al., 2008; Snyder et al., 2012). Moreover, neuronal activity in the locus coeruleus precedes activity in the prefrontal cortex that is triggered by a CS (Snyder et al., 2012). Our observations that propranolol prevented extinction in the AAA paradigm is in line with the likelihood that noradrenaline release from the locus coeruleus is required in circumstances that require enhanced attentional focus and an associated change in behavioral strategy, as proposed by others (Bouret and Sara, 2005; Yu and Dayan, 2005; Dayan and Yu, 2006). In addition, our findings suggest that this kind of neuromodulation is mediated by noradrenaline acting on β-adrenergic receptors. This in turn may enable qualitative control over extinction learning whereby, under specific circumstances, attentional focus is optimised when extinction learning should take place under subtle (constant context) conditions. In line with this, a role for noradrenaline in the neuronal encoding of prediction errors has been proposed (Schultz and Dickinson, 2000). This would support, for example, attentional focus towards and the registration of subtle changes in environmental conditions that could facilitate extinction learning.

## Concluding Remarks

In conclusion, the findings of this study indicate that in an appetitive learning task that includes low reward probability, antagonism of β-adrenergic receptors impairs extinction in the absence of a context change (AAA paradigm), but does not affect extinction that is supported by a change of context (ABA paradigm). The inhibition of extinction that occurred in the AAA paradigm suggests that NA modulation of attentional focus is an important factor for the extinction of appetitive experience. Recent studies conducted in the context of reconsolidation blockage have indicated that propranolol prevents the reconsolidation of emotional memories (Kindt et al., 2009; Schwabe et al., 2012). These studies raise hope for the usage of propranolol as a potential treatment for post-traumatic stress disorder (Pitman and Delahanty, 2005). However, other studies reported that propranolol impairs fear extinction in humans, especially at a cognitive level (Bos et al., 2012). Taken together, with findings obtained under non-emotive/non-fearful conditions, this suggests that the effects of beta-blockade might be harmful, rather than beneficial, if extinction takes place in an appetitive context, and if cognitive rather than affective changes are desired.

## Conflict of Interest Statement

The authors declare that the research was conducted in the absence of any commercial or financial relationships that could be construed as a potential conflict of interest.
